# Switchable Polarization in Mn Embedded Graphene

**DOI:** 10.1038/s41598-018-22583-4

**Published:** 2018-03-14

**Authors:** Mohammad Noor-A-Alam, Hamid Ullah, Young-Han Shin

**Affiliations:** Multiscale Materials Modeling Laboratory, Department of Physics, University of Ulsan, Ulsan, 44610 Republic of Korea

## Abstract

Graphene, despite its many unique properties, is neither intrinsically polar due to inversion symmetry nor magnetic. However, based on density functional theory, we find that Mn, one of transition metals, embedded in single or double vacancy (Mn@SV and Mn@DV) in a graphene monolayer induces a dipole moment perpendicular to the sheet, which can be switched from up to down by Mn penetration through the graphene. Such switching could be realized by an external stimuli introduced through the tip of a scanning probe microscope, as already utilized in the studies of molecular switches. We estimate the energy barriers for dipole switching, which are found to be 2.60 eV and 0.28 eV for Mn@SV and Mn@DV, respectively. However, by applying biaxial tensile strain, we propose a mechanism for tuning the barrier. We find that 10% biaxial tensile strain, which is already experimentally achievable in graphene-like two-dimensional materials, can significantly reduce the barrier to 0.16 eV in Mn@SV. Moreover, in agreement with previous studies, we find a high magnetic moment of 3 *μ*_B_ for both Mn@SV and Mn@DV, promising the potential of these structures in spintronics as well as in nanoscale electro-mechanical or memory devices.

## Introduction

Graphene—one atomic layer of C atoms arranged in a hexagonal lattice with many unique and extraordinary properties—has been an object of intense research activities that might revolutionize next generation nano scale devices^1–3^. Despite its unique electronic properties due to Dirac points in the corners of the Brillouin zone, it is intrinsically non-polar due to its centrosymmetric crystal structure, which hinders its application in nanoelectromechanical systems. However, recent experimental and theoretical works have confirmed that polarity—consequently piezoelectricity—can be engineered by breaking the inversion symmetry in various ways; such as creating holes of the right symmetry that results in a strain gradient under a uniform stress^4^, adsorption of foreign atoms on the surface^5^, or chemical modification like oxidization^6^, or hydrogen and fluorine codecoration on the surface^7–9^. Note that creating holes with right symmetry is experimentally challenging, whereas chemical modifications drastically change the electronic properties of graphene^7–9^. On the other hand, due to the weak interaction between adatoms and graphene^5^, adsorbed foreign atoms have tendency to migrate on the surface with a low energy barrier of order of 0.1–0.3 eV. At room temperature or high actuation frequency, such low migration barrier may cause the adatoms mobile on the surface or even may lead to desorption, causing potential failures in practical applications. Moreover, polarization switching—a signature of ferroelectricity and an essential property for memory devices—might not be feasible in such structures, because penetration barrier for adatoms through the graphene is quite high, and rotating C-H/F bonds in codecorated graphene could require high energy^8^.

High-performance ferroelectric materials at nanoscale hold a great potential for miniaturization of devices, high-density nonvolatile memory storage, and discovery of exotic physical phenomena^10,11^. However, due to depolarization field, arising from the uncompensated charges at the interfaces, polarization perpendicular to the film surface in ultra thin ferroelectric films is suppressed, which stems a critical thickness for ferroelectricity^10,11^. Interestingly, a new class of two-dimensional (2D) honeycomb binary monolayers has been reported very recently^12^. Those free standing buckled monolayers are theoretically predicted to be stable with a high polarization. However, those monolayers are yet to be realized experimentally. Moreover, a robust ferroelectricity perpendicular to its plane has also been predicted in a distorted 1T-MoS_2_ monolayer under a spontaneous symmetry breaking, although it has not been experimentally conformed yet^13^. Promisingly, experimental observation (followed by the theoretical prediction) of piezoelectricity in MoS_2_ membranes with the odd number of layers has already been reported for nano-electro-mechanical systems (NEMS)^14,15^. As a consequence of device miniaturization, here it should be mentioned that molecular switches^16^ –mainly metallorganic phthalocyanine molecules could become an alternative paradigm to the conventional ferroelectric materials for data storage and logic circuits. However, for practical realization of such switch, it is essential to assemble these molecules on a surface without forming any strong bonds^16^. Under those circumstances, as periodicity does not hold perpendicular to the graphene sheet, hence engineering a switchable dipole moment in graphene in a way that is experimentally feasible might pave the way to open up a new possibility of a class of nanoscale “dipoletronics”^13^ devices.

On the other hand, the effect of localized magnetic impurities in graphene on its electronic and induced magnetic properties have been extensively studied both experimentally and theoretically in the context of spintronics and unconventional Kondo effect^17–20^. Realization and control over the high-spin state of an isolated single atom magnet are ultimate goals for spintronics, which may lead the magnetic storing devices to super storing capacity^17,19^. As a result, a considerable body of theoretical as well as experimental works has been devoted on an isolated transition metal (TM) atom in a graphene sheet, which is considered as an ideal situation because of non-magnetic graphene^17–19,21^. In this regard, recent experimental demonstration by electron spectroscopy on the realization of the single atom spin state at atomic defects in graphene is a great achievement^17^. Such structures have been studied theoretically as well^19,20^. However, electric polarity of such structures originated from the fact that TM atoms at defect sites locally break the flatness of graphene, has not been investigated yet.

In this study we investigate the polarity of a Mn atom embedded in a single or double vacancy of a graphene sheet. We find a considerably large dipole moment, which is comparable with other 2D polar materials^8,9,12,13,22–25^. Moreover, we also estimate the energy barrier for dipole switching from up to down by Mn penetration throughout the graphene. The barrier for Mn@SV penetration is found to be high, however reasonably low for Mn@DV. Note that the impenetrability throughout pristine graphene has been already shown for helium and hydrogen atoms experimentally^26^ as well as theoretically^27^. However, to tune the penetration barrier for Mn@SV, we propose a mechanism that employs a biaxial strain. We show that 10% biaxial tensile strain, which could be experimentally achievable for graphene like 2D materials^28,29^, can significantly reduce the barrier indicating a way to switch the dipole moment as such structures are already experimentally available^17^. We also study the effect of substrate on our systems, for the purpose we consider a layer of graphene underneath the Mn-embedded system. We find a significant change in the energy barrier while no large change in the magnetic moment. Hence we expect that our findings may stimulate more studies to find an experimental way to realize such switching of dipole moment in TM embedded graphene along side with its magnetism, and may find applications in nanoscale memory devices.

**Figure 1:**
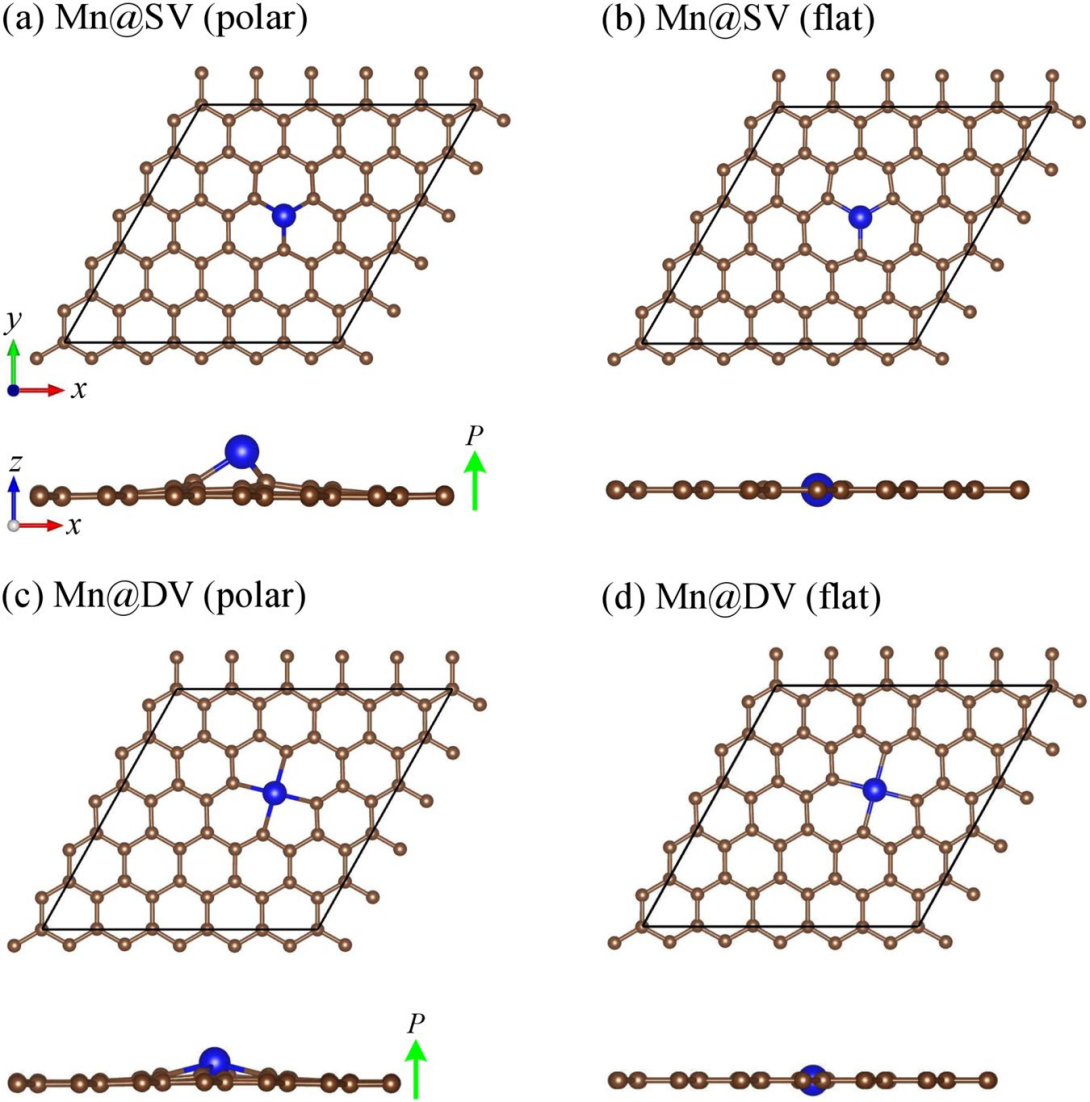
Optimized structures are shown. Brown and blue balls represent the carbon and Mn atoms, respectively. The considered unit cells are shown by black lines. Upward green arrows represent the out-of-plane polarizations directing from the graphene sheet to the Mn atom.

In addition to the previously studied configuration (in this paper which is labeled as’polar’) with a Mn atom outwardly protruded from the graphene surface^19,20^, we consider the flat configuration where a Mn atom is located in the same plane of carbon in the graphene. Optimized configurations are shown in Fig. 1, and the optimized structural parameters are given in Table 1. From binding energy (*E*_*b*_), we find that polar configurations are always more stable than the flat ones because of the larger atomic radius of Mn atom compared to C. In agreement with the previous studies^19,20^, the height (*h*) of the Mn atom over the graphene plane significantly reduces from 1.44 Å for Mn@SV to 0.74 Å for Mn@DV due to the fact that the DV opens a larger space under the Mn atom. However, even the flat configurations have significantly large binding energies (shown in Table 1), indicating that the Mn atom remains bonded with C atoms and may require a large energy to escape from the vacant site. Note that TM atoms adsorbed on the pristine graphene are reported to have binding energy of 0.2–1.5 eV with a low migration barrier in the range of 0.2–0.8 eV^19^. On the other hand, in agreement with previous studies^19,20^, we also find that both Mn@SV and Mn@DV possess a magnetic moment of about 3 *μ*_B_; whereas an isolated Mn atom has a magnetic moment of 5 *μ*_B_. Moreover, our calculations show that the magnetic moment of Mn@SV remains 3 *μ*_B_ even for the flat configurations. For Mn@DV, it slightly increases to 3.41 *μ*_B_ from 3 *μ*_B_. Since the magnetism in transition-metal embedded graphene is already well-studied^17–20^, we rather only focus on the polarity of such magnetic configurations.

**Table 1:** Optimized lattice parameters *a* (Å), binding energy *E*_*b*_ (eV/Mn), bond length between Mn and carbon *l*_Mn−*C*_ (Å), height *h* (Å) of the Mn on graphene, magnitude of electric polarization *P*_*z*_ (pC/m), and magnetic moment *M* (*μ*_B_) are tabulated.

Structures	*a*	*Eb*	*l*Mn − C	*h*	*Pz*	*M*
Graphene	12.34	—	—	—	—	—
Mn@SV (polar)	12.37	6.51	1.83	1.44	3.95	3.00
Mn@SV (flat)	12.49	3.90	1.72	—	—	2.97
Mn@DV (polar)	12.33	4.06	1.98	0.74	1.71	3.06
Mn@DV (flat)	12.36	3.83	1.96	—	—	3.41

**Figure 2:**
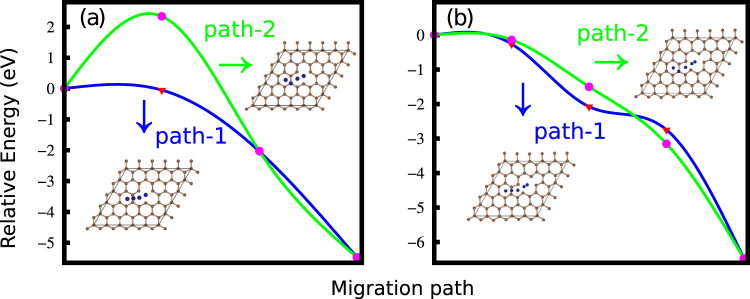
The migration of a Mn atom across the graphene with (**a**) single and (**b**) double vacancies. The migration paths are shown in the insets, which are indicated by the arrows.

We also investigate the migration of a Mn atom across the graphene with single or double vacancy. In experiments, initially a Mn atom can be adsorbed anywhere on the graphene with SV or DV as metal atoms are deposited on the pretreated surface by using thermal evaporation. Here it should be mentioned that usually high energy electron beam irradiation is used in experiments to create such defects in graphene^17^. Figure 2 shows the change in energy as the Mn atom migrates from the initial adsorption site to the neighboring vacant site. For both SV and DV, we consider two paths of migration. Starting from the hollow site, the Mn atom migrates either over the C-C bond (path-1) or over the C atom (path-2). Interestingly, our nudged elastic band (NEB) method calculations (shown in Fig. 2) show barrierless migration along path-1 for both SV and DV, indicating spontaneous formation of Mn embedded graphene. Irrespective to the initial absorption site, the Mn atom will go to the nearest vacant site. For the reverse process, a significantly large energy (shown in Fig. 2) is required for the Mn atom to escape from the embedded site. In other words, the Mn atom will be trapped in the vacant site.

As the polar configurations are energetically more favorable than the flat configurations, here we investigate the polarity of such polar configurations. First, we compute the charge transformation between Mn and graphene, employing Bader charge analysis^30^. A significant charge transformation of 0.90 |*e*| from Mn to graphene is found for both polar configurations, indicating a local redistribution of electrons and consequently a local dipole moment directing from graphene to Mn. Note that such charge transformation and dipole moment are also found for metal adatoms on perfect graphene. For instance, Mn atoms adsorbed at hollow sites of perfect graphene^31^ transfer 0.84 |*e*| to graphene.

As adsorbed Mn atoms in ‘polar’ configurations break the flatness of graphene as well as redistribute electrons by charge transformation, an electric dipole moment has induced. The electric polarization *P* (dipole moment per unit area due to dimensionality) perpendicular to the graphene sheet is computed as

1 $${P}_{z}=\frac{e}{A}(-{\int }_{0}^{{L}_{z}}\rho (z)zdz+\sum _{i=1}^{N}{Z}_{i}{z}_{i}),$$

where *ρ*(*z*) is the planar-averaged electron density along *z*-direction, *Z*_*i*_ is the net atomic number of *i*-th ion with *z*_*i*_ being the *z* coordinate in the unit cell of area *A*, *L*_*z*_ stands for the length of a unit cell along the *z*-direction, and *e* is the absolute value of an electronic charge. A similar definition was used in our previous studies^8,22,23^. The calculated values are shown in Table 1.

Note that the lack of band gap in our configurations does not prohibit an out-of-plane electric polarization, as conductivity is only in the plane. Perpendicular to the graphene sheet, the configurations can be considered as insulating. Such a unique property of 2D materials was also used in other previous studies^5^. Now we show that a reasonably larger polarization directed from the graphene to Mn is found in Mn@SV in comparison with Mn@DV; mainly because the height of the Mn on the graphene is significantly reduced in Mn@DV. Obviously being completely flat,’flat’ configurations exhibit no out-of-plane dipole moment. Note that despite the low dimensionality of our configurations, such polarizations down to the nanoscale are not negligible, in fact comparable with others. We first compare the polarizations with other 2D polar materials. Our estimated values are quite comparable with the values for group IV binary monolayers (SiGe, SiSn, and GeSn), however slightly lower than the estimated values for III-V binary monolayers (AlSb and GaAs)^12^. Note that the polarizations in hydrogen and fluorine co-decorated graphene^8^, silicene^23^, and *h*-BN^22^ are an order of magnitude larger. Now, considering the height of our configurations as the thickness (shown in Table 1), we may express our values in three-dimensional (3D) units. The polarizations in 3D units become roughly 2.75 *μ*C/cm^2^ and 2.31 *μ*C/cm^2^ for Mn@SV and Mn@DV, respectively, which are an order of magnitude larger than the value (0.28 *μ*C/cm^2^) predicted for ferroelectric polarization in distorted MoS_2_ monolayer^13^. However, the polarizations are about four times lower than the polarization in ferroelectric polymers (for example, in P(VDF-TrFE)^32^ the polarization is about 8 *μ*C/cm^2^). We also find that our estimated polarizations are also comparable to those of wurtzite semiconductors such as GaN (3.4 *μ*C/cm^2^) or InN (4.2 *μ*C/cm^2^)^33^, and an order of magnitude larger than the measured values in 2D freely suspended ferroelectric smectic-C films^24^ and nematic liquid crystal monolayer^25^. For further comparison, our estimated values are slightly larger than the polarizations of improper ferroelectrics^34^ (La,Pr)Al_2_O_6_ (1.80 *μ*C/cm^2^) and (Ce,Pr)Al_2_O_6_ (1.78 *μ*C/cm^2^); moreover, are in the same order of magnitude with the polarizations of hexagonal *R*MnO_3_ (where *R* stands for rare earth elements) with a typical value of a few *μ*C/cm^2^ (for example, 5.6 *μ*C/cm^2^ for multiferroic YMnO_3_)^35^. Also, the polarization of about 5 *μ*C/cm^2^ in multiferroic double-perovskite Bi_2_NiMnO_6_ structure^36^ is twice larger than that in our configurations.

**Figure 3:**
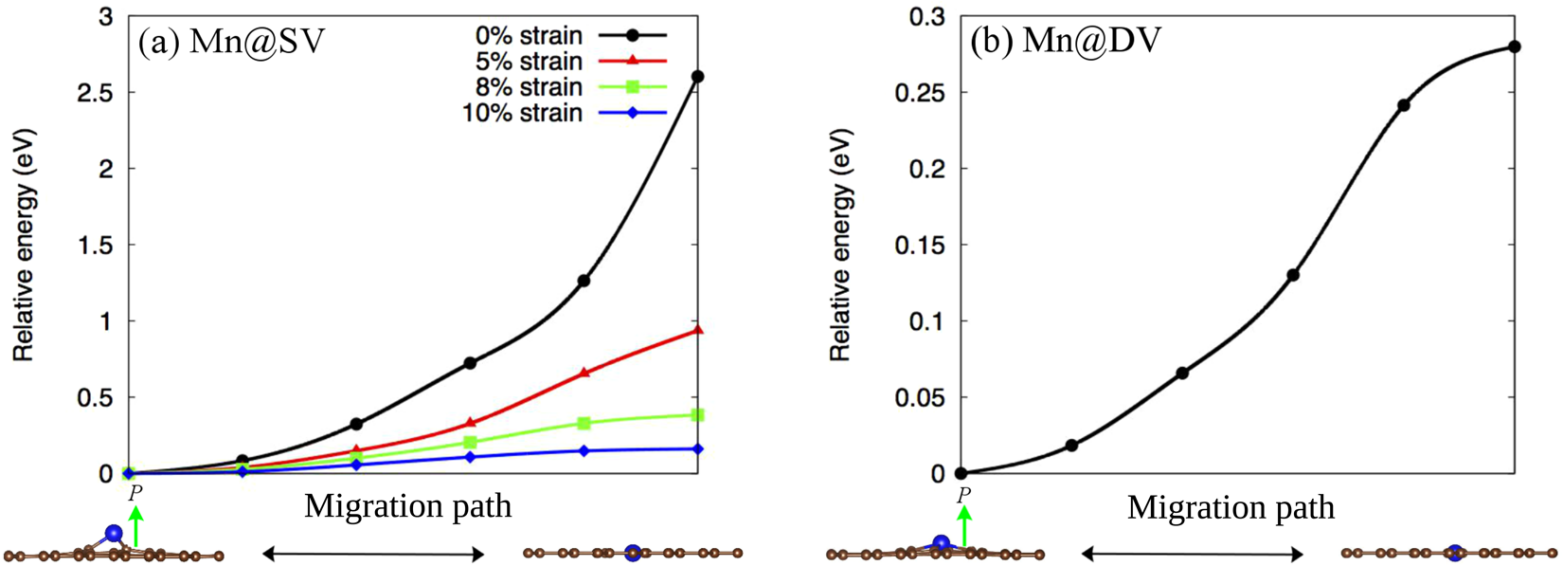
Energy barriers of Mn@SV and Mn@DV from the polar configurations to the flat configurations.

Now we turn our attention to the possibility of the polarization switching, where polarization changes sign by the penetration of Mn throughout the graphene sheet. As the up and down polarizations states–where the Mn atom is right above or under the vacancy-site–are symmetric with respect to the flat state, therefore we only consider the barrier path from up to flat configurations. In order to estimate the energy barrier for a dipole moment switching between initial polar to final flat configurations, we perform the NEB calculations with four images between the initial (polar) and final (flat) configurations (shown in Fig. 3). The energy barrier for Mn@SV is found to be 2.60 eV, which is quite high and indicates the impenetrability of the graphene sheet. However, interestingly the barrier for Mn@DV (0.28 eV) is rather significantly lower, because of the larger hollow space in the graphene due to DV. Also note that the lattice parameters of the flat Mn@SV configuration have significantly increased (about 1% with respect to its polar configuration), whereas for flat Mn@DV, indicating its lower penetration barrier, a minor change (only about 0.24%) in lattice parameters is observed (shown in Table 1). The barrier height is quite comparable to the heights estimated (in the range of 0.1–0.3 eV) for the 2D binary compounds^12^. Here we emphasize that such dipole switching could be considered as analogous to the molecular switching^16^ where a molecular state can be switched from one to another under an external stimuli usually through a scanning probe microscope with a threshold voltage of around 2–4 eV. Hence, employing the similar technique, even switching in Mn@SV could be a possibility. However, tuning the barrier may introduce an additional control over the switching between configurations. We find that a biaxial tensile strain can be an effective way to tune the barrier, as the space beneath the Mn increases due to the strain. Figure 3 shows that the barrier indeed significantly reduces to 0.16 eV at 10% strain. Note that experimentally such strain could be obtained, as graphene has been already found to hold a large strain up to about 12%^28,29^. To this end, it should be noted that energetically non-equivalent states might arise in the presence of a substrate. Hence for real device applications, we suggest equivalent electrodes for instance top and bottom graphene electrodes.

**Figure 4:**
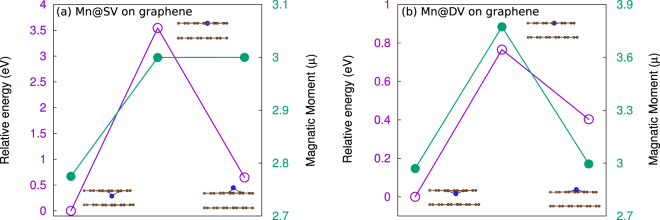
Energy barriers and magnetic moments for (**a**) Mn@SV on graphene and (**b**) Mn@DV on graphene.

Finally, to observe the effect of a substrate, we consider a layer of graphene underneath the Mn-embedded one. In real experiment, graphene can be used as a buffer layer between a substrate and Mn-embedded graphene. We find that up and down polarization states are not energetically equivalent on graphene. The down state becomes more favorable than the up state, as shown shown in Fig. 4. The down polarization state is 0.5 eV (0.4 eV) lower in energy with respect to the up polarization configuration of Mn@SV (Mn@DV). As a result, the switching barrier from down to up 3.52 eV (0.78 eV) is higher than that from up to down 3.02 eV (0.38 eV). Interestingly, in comparison with free standing Mn-embedded graphene (See Fig. 3), the switching barrier significantly increases for both Mn@SV (0.92 eV) and Mn@DV (0.10 eV)on graphene (See Fig. 4).

Additionally, a significant change in electric dipole moment during the switching has also been observed in the presence of graphene. Now up and down polarization states have not only different energies but also different electric dipole moments. Compared to free standing Mn@SV (0.32 |*e*|Å), the presence of graphene decreases the electric dipole moment of the down state (0.18 |*e*|Å), although the dipole moment of the up state (0.29 |*e*|Å) has changed a little. On graphene the dipole moment of the down state of Mn@DV (0.31 |*e*|Å) is larger than that of free standing Mn@DV (0.14 |*e*|Å). However, the dipole moment of the up state (0.14 |*e*|Å) remains the same as the value of free standing. On the other hand, magnetic moments remain almost the same during the electric polarization switching (See Fig. 4). Nevertheless, we find an interesting correlation between change in electric and magnetic dipole moments on graphene. We notice a slight change in both electric and magnetic moments only for down electric polarization states in both Mn@SV and Mn@DV due to the presence of graphene substrate. Here it should be mentioned that substrates have also been found to play a vital role in electric and magnetic dipole moments of molecular switches^16^. Here it should be noted that Mn embedded graphene can be an ideal structure for realizing tunable unconventional Kondo effect by an applied gate voltage as proposed in ref.^18^. Our proposed structure can also be used as a spin injector in a graphene spin-current demultiplexer (GSDM)^37^. Also note that under a gate voltage substrates can affect the electric and magnetic moments as electric field can be a good way to control the charge transfer between Mn@Gr and the substrate. Additionally, it will be interesting to know the effect of substrates for the proposed gate controlled Kondo effect^18^ and GSDM^37^, which is beyond the scope of this paper and left for the further studies.

In summary, we demonstrate a practical route to engineering switchable polarization in non-polar graphene.The switching barriers are in the range of the usual barriers of molecular switching. Furthermore, we show that a biaxial tensile strain that is already shown to be experimentally achievable can significantly reduce the switching barrier providing an additional control over the switching. Indicating a possible route of realizing such metal embedded graphene, our calculations show that Mn atoms migrate to the nearest vacant sites spontaneously irrespective to the initial absorption sites. More interestingly, we also show that a substrate can significantly change polarization switching barrier as well as electric and magnetic dipole moment. Although here we only investigate the Mn in graphene, we emphasize that our findings can easily be extended for the other TM elements. Since it has been already demonstrated that the data storage is possible in molecular switches, we believe that our results may stir more research activities as well as find applications in graphene based data storage nano devices.

Our first-principles calculations are performed by the spin-polarized density functional theory using projector augmented wave (PAW) potentials to describe the core electrons and the generalized gradient approximation (GGA) of Perdew, Burke, and Ernzernhof (PBE) for exchange and correlation as implemented in the Vienna Ab initio Simulation Package (VASP)^38–40^ based on a plane-wave basis set. Graphene supercells (5 × 5 graphene unit cells) containing 49 (for Mn@SV) or 48 (for Mn@DV) C atoms with a Mn atom are used to simulate the isolated single Mn atom on graphene. As periodic boundary conditions are imposed in all three directions, the distance between two neighboring periodic images in the normal direction is kept larger than 20 Å in order to avoid interactions between periodic atoms. A large enough cutoff energy of 500 eV for the plane-wave expansion is used in all calculations to avoid the basis set incompleteness. All structures are fully relaxed until the Hellmann-Feynman forces on all the atoms are less than 10^−3^ eV/Å. The geometry optimization of configurations is carried out employing the conjugated gradient technique. The criterion for the total energy is set as 10^−7^ eV. The Brillouin zone is sampled with a Γ-centered *k*-point mesh of 7 × 7 × 1 for geometry optimizations, while a denser grid of 15 × 15 × 1 is used for charge density calculations. Dipole correction^41^ is used to cancel out the artificial electric field that arises from the periodic boundary conditions in polar surface calculations. For Mn embedded graphene on graphene, the Grimme’s DFT-D2 method^42^ is used to capture the van der Waals interactions.

Calculations to estimate the penetration barrier of Mn throughout the graphene are performed using the nudged elastic band method (NEB)^43^ with four images along the penetration path. The binding energy (*E*_*b*_), an essential quantity closely related to the binding strength between Mn and graphene, is calculated as *E*_*b*_ = *E*_G*r*−*SV*/*DV*_ + *E*_Mn_ − *E*_Mn@SV/DV_; where *E*_Mn@SV/DV_ is the total energy of a Mn atom in SV or DV of graphene, *E*_Gr−SV/DV_ is the total energy of the graphene sheet with a SV/DV, and *E*_Mn_ is the energy of an isolated Mn atom. A positive value of *E*_*b*_ with larger amplitude means that the configuration (Mn@SV/DV) is thermodynamically favorable.
